# Interventions Promoting Work Engagement and Reducing Turnover of Newly Graduated Nurses: A Systematic Review

**DOI:** 10.1002/nop2.70434

**Published:** 2026-02-15

**Authors:** Laura al‐Hello, Jenni Ervasti, Toni Haapa, Ville‐Pekka Sorsa, Hanna‐Leena Melender

**Affiliations:** ^1^ Division of Ear, Nose and Throat Diseases, Head and Neck Center Nursing Research Center Helsinki University Hospital and University of Helsinki Helsinki Finland; ^2^ Finnish Institute of Occupational Health, University of Helsinki Helsinki Finland; ^3^ Nursing Research Center Helsinki University Hospital and University of Helsinki Helsinki Finland; ^4^ Faculty of Social Sciences University of Helsinki Helsinki Finland; ^5^ Research Unit of Health Sciences and Technology University of Oulu Oulu Finland

**Keywords:** nurses, personnel turnover, programme evaluation, systematic review, work engagement

## Abstract

**Aim:**

To evaluate the effectiveness of interventions to enhance and maintain organisational commitment, work engagement and retention and to reduce burnout and turnover of newly graduated nurses (NGNs).

**Background:**

Nurses leave their profession at a rate of 5%–18% worldwide. NGNs transferring from being a nurse student to a registered nurse may face significant stress and difficulties. To support a successful transition and reduce turnover rates among new graduates, it is necessary to identify effective interventions to enhance and maintain organisational commitment, work engagement and retention of NGNs and to reduce their burnout and turnover.

**Methods:**

The systematic review focused on newly graduated nursing professionals having worked within clinical practice at most 6 months after their graduation, in any social or health care setting. We excluded studies focusing solely on nursing assistants, licensed practical nurses, advanced nurse practitioners, or clinical nurse specialists. Of the interventions, residency programmes were excluded. Cochrane Library, CINAHL, MEDLINE, Scopus, the Joanna Briggs Institute and Medic were searched with a timeline from January 2012 to February 2023. Quality appraisal of the original studies was performed using specific criteria for each study design. The results of the original studies were synthesised narratively. Effect size was estimated with Cohen's *d* (*d*).

**Results:**

The review included three moderate to good quality original studies with 566 participants in total: a randomised controlled trial, a non‐randomised trial and a cohort study. Two interventions based on one‐to‐one mentoring strategy (*d* = −0.18) and 10‐minute preceptor model using educational strategies (*d* = −0.58) showed small or medium *effects towards decreased turnover intentions.* Psychological measures of work engagement, burnout or organisational commitment had not been addressed as an outcome in any of the studies.

**Conclusion:**

We found very few good‐quality studies examining interventions to promote NGN's staying at work. Of the three studies, two showed an association with lower turnover. More research with high‐quality study designs is needed. The evaluations should include cost–benefit analysis.

**Implications for Nursing Management:**

The current evidence is insufficient to make recommendations for nursing management on interventions to promote NGN's staying at work. Thus, further research is needed to build evidence‐based interventions.

**Registration:**

PROSPERO Registration CRD42022328406

## 1 Background

Globally, there was a shortage of nearly 6 million nurses in 2018 (WHO 2016). One of the causes is the high rate of turnover among newly graduated nurses (NGNs). In 2013, the World Health Organization (WHO) estimated that 40% of healthcare workers may quit their professions within 10 years, and that there will be a 15 million global shortfall of health workers by 2030 (WHO 2016). Nurses leave the profession at a rate of 5%–18% worldwide (Lee et al. 2017). A growing share of nurses are considering switching careers, even if they aren't actively seeking work. Moreover, fewer students are graduating from health education programmes (Halter et al. 2017; von Hoeve et al. 2019).

The shortage of competent nurses contributes to decreased quality of care and, from time to time, decreases in patient safety. It also creates problems for nursing management. On the organisational level, human resource management is key for reducing voluntary turnover rates in all work settings. There is also some evidence that nurse managers' leadership styles have an influence on reducing turnover (Drennan and Ross 2019). Management can effectively contribute to nurses' organisational commitment and the profession (Brook et al. 2019).

NGNs transferring from being a nurse student to a registered nurse may face significant stress and difficulties. Doughty et al. (2018) concluded that it is important to analyse the causes of new nurses' turnover and departures from the profession as well as find solutions to boost and sustain their organisational commitment, work engagement and retention to facilitate a smooth transition and lower turnover rates after immediate employment. In a longitudinal study by Rudman and Gustavsson (2011), almost every fifth NGN reported extremely high levels of burnout at some point during their first years after graduation. Changes in burnout levels were accompanied by intention to leave the profession. NGNs have reported that perceived lack of respect, poor management, lack of training and prospects and poor salary lead some nursing professionals to consider leaving their work (Flinkman 2014), but higher work engagement among nurses has been associated with both higher job satisfaction and higher patient satisfaction (Simone et al. 2018). Finding ways to improve NGNs' job satisfaction, engagement and job retention is thus crucially important (Lee et al. 2017).

Empowering workplace structures, such as opportunities for learning, growth and advancement as well as support and sufficient resources can buffer the shock of working life realities faced by NGNs entering work. The theory of structural empowerment proposes that structural power improves work engagement and organisational commitment indirectly through the psychosocial work environment. This theory has been tested among NGNs, and some evidence supporting paths from structural empowerment to a good psychosocial work environment, high work engagement/less burnout and higher organisational commitment was observed, thus providing evidence linking Kanter's theory with Maslach and Leiter's theory of work engagement (Maslach and Leiter 2017; Cho et al. 2006).

Based on the hypothesis that it is possible to enhance structural empowerment, the aim of this systematic review was to evaluate the effectiveness of interventions to enhance work engagement and reduce burnout, and to improve organisational commitment and reduce turnover of NGNs. The purpose is to support nursing leaders' and managers' efforts in retaining their nursing workforce by synthesising evidence on effective interventions. A previous systematic review by Brook et al. (2019) evaluated characteristics of successful interventions to reduce turnover and increase retention of early career nurses. The studies included in their review were published between 2001 and April 2018. The current systematic review includes studies published between 2012 and February 2023. Although there is some time‐related overlap between the two reviews concerning the years 2012–2018, there is no content‐related overlap since the inclusion criteria have not been the same. For example, interventions where retention could be influenced by financial resources, such as residency programmes, were not included in our review, while the review by Brook et al. (2019) included those as well. Moreover, this systematic review also addresses organisational commitment, work engagement and burnout as outcomes, whereas the review by Brook et al. (2019) doesn't cover these outcomes.

## 2 Methods

This systematic review was conducted and reported in accordance with the PRISMA (Preferred Reporting Items for Systematic Review and Meta‐Analyses) guidelines (Page et al. 2021) and registered in PROSPERO (International prospective register of systematic reviews).

### 2.1 Eligibility Criteria

The review focused on newly graduated nursing professionals such as registered nurses, public health nurses, paramedics and midwives. There were no limitations regarding the age, gender or ethnicity of participants. For the purposes of this review, NGNs were defined as nurses having worked within clinical practice at most 6 months after their graduation. The review considered studies carried out in any social or health care setting and country where NGNs work, provided that the nurses have less than 6 months of experience in clinical nursing practice.

The review excluded studies focusing entirely on nursing assistants, licensed practical nurses, advanced nurse practitioners or clinical nurse specialists. Of the interventions, residency programmes were excluded from this review, because the focus was on interventions conducted in situations where the NGNs have been working full‐time in direct patient care with a full salary.

### 2.2 Search Strategy and Data Quality Appraisal

The search was conducted from the following databases: Cochrane Library, CINAHL, MEDLINE, Scopus, the Joanna Briggs Institute and the Finnish health sciences database Medic. The original search involved a timeline from January 2012 to April 2022, and the search was updated in February 2023. The search terms were the same across all six databases both times. Appropriate subject headings and/or keywords were used (Table S1). Two academic librarians were consulted to assist in constructing search terms and search strategies. The search strategy was based on a PICO structured question, including NGNs as P (population), interventions to enhance and maintain organisational commitment, work engagement or work retention, or to reduce burnout or turnover as I (intervention), RCT studies, non‐randomised trials and cohort studies as C (comparison), and organisational commitment, work engagement, retention, burnout and turnover as O (outcome). In addition, the reference lists of the identified original studies were checked for additional relevant studies.

### 2.3 Study Selection and Quality Appraisal

**FIGURE 1:**
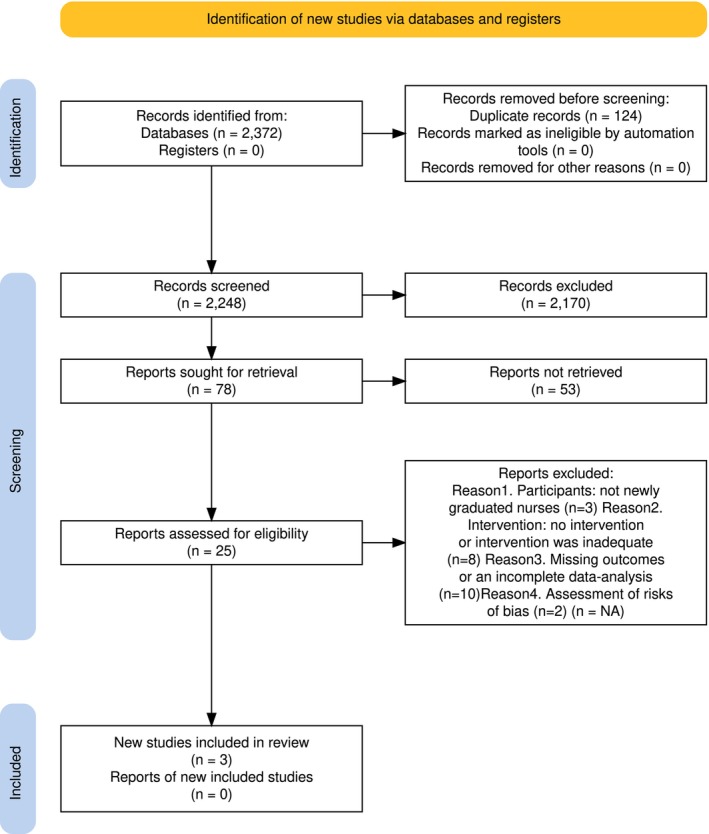
Flow diagram (Page et al. 2021) of the literature search and selection process.

All search results were downloaded and saved in the main author's own datafiles. Duplicate records were removed. Titles, abstracts and full texts were screened by two independent reviewers to check the eligibility for inclusion against the inclusion criteria. Disagreements between the two reviewers were resolved with discussion. The reasons for excluding studies were as follows: the participants were not NGNs, the study did not include any intervention, the intervention was inadequate, there were missing outcomes, or the data analysis was incomplete. The selection process is illustrated in Figure 1. Rayyan's (web version) Systematic Review Platform (Ouzzani et al. 2016) was used in the screening of the titles and the abstracts.

Two independent reviewers assessed the search outcome for methodological validity prior to inclusion in the review using standardised critical appraisal instruments, which were Cochrane risk of bias tools for randomised trials for one study (Cochrane 2020), NIH NHLBI Quality Assessment Tool for quasi‐experimental design for three studies (NIH 2021) and Cochrane Tool to Assess Risk of Bias in Cohort Studies for one study (Cochrane 2019).

### 2.4 Data Synthesis

The results or the original studies were synthesised narratively. The main author extracted information on the development, aim, content and duration of each intervention from the original articles. To present the original studies, information on the purpose of the study, setting and participants, outcomes, data collection and data analysis were extracted (Table 1). The main outcomes included in the review were organisational commitment, work engagement, retention, burnout and turnover. No additional outcomes were analysed. Statistical analyses were conducted using the Python programming language (version 3) (Python Software Foundation 2023). The standard for assessing the size of effect sizes (Cohen's *d*) was as follows: small (*d* = 0.2), medium (*d* = 0.5) or large (*d* ≥ 0.8) (Sullivan and Feinn 2012).

**TABLE 1:** Description of the studies included in the review.

Authors (year), country, title, study design, quality of the study	Purpose of the study and development, aim, content and duration of the intervention	Setting, participants and follow‐up time^a^	Measures and method of statistical analysis
Concilio et al. (2021), USA A prospective, randomised controlled trial Quality: MODERATE	*Purpose of the study*: To determine the impact of a 6‐week digital intervention on newly licensed graduate nurses' (NGLN) levels of perceived stress, resiliency, social support and intention to leave their current job, organisation and profession in the first year of hire *Development of the intervention*: Supportive text messages were created based on the work of the Social Support Behavioral Code (SSBC). The contents of the text messages were validated (I‐CVI) by a panel of five expert RNs with two rounds *Aim of the intervention*: To impact NGLN's stress, resilience, sense of support and intention to leave *Content of the intervention*: A 6‐week digital intervention. Experimental group received supportive text messages based on SSBC while control group received text messages of medical facts that were not meant to support or correlate with SSBC. Medical facts were selected based broadly on fundamental knowledge previously taught in undergraduate coursework *Duration of the intervention*: 6 weeks	*Setting*: Two urban health care systems located in western Pennsylvania and southern California, USA *Participants*: Newly licensed graduate nurses (*n* = 21) who were in their first year of hire The experimental group (*n* = 10) received the supportive text message intervention The control group (*n* = 11) received text messages of medical facts *Follow*‐*up time*: The follow‐up measurements with the survey were conducted simultaneously during the implementation of the intervention at weeks 3 and 6; no follow‐up after the intervention had finished	*Work engagement*: — *Burnout*: — *Organisational commitment*: — *Retention*: — *Turnover intentions*: A 3‐item questionnaire measuring NLGNs' intention to leave their current job, organisations and the nursing profession (Developed for this study) *Turnover*: — *Analysis method*: Bayesian *t*‐test. A Bayesian factor (BF) greater than 1 indicates evidence to support varying degrees that there is no difference between groups; a BF less than 1 indicates there is a difference between the groups
Hu et al. (2015), Taiwan, China Cohort study A repeated‐measures design study, intervention Quality: GOOD	*Purpose of the study*: To develop and evaluate the effects of a 10‐minute preceptor (10MP) model for assisting NGNs in their professional development and increasing their retention in hospitals *Development of the intervention*: Intervention was developed by the research team based on the five‐step micro‐skill model of clinical teaching *Aim of the intervention*: To guide preceptors to dedicate 10 min, twice per day, to structurally communicate, interact and discuss problems and issues with the NGNs during the initial 3 months of orientation *Content of the intervention*: Training programme included three phases: 1. Informing preceptors when to interact with an NGN 2. Informing preceptors of what they should accomplish in a day (teaching points), including setting learning goals and learning content, showing concern and providing support and feedback to NGNs 3. Guiding a preceptor to ask NGNs specific questions twice per day (tasks) *Duration of the intervention*: 3 months	*Setting*: A Teaching Hospital, Taipei City, China. *Participants*: NGNs (*n* = 107) The experimental group (*n* = 54) received a 10‐minute Preceptor Model (10PM) intervention The control group (*n* = 53) received a traditional preceptor model (TMP) as an intervention *Follow*‐*up time*: The follow‐up measurements were conducted simultaneously during the implementation of the intervention at day 7 and at months 1, 2 and 3; no follow‐up after the intervention had finished	*Work engagement*: — *Burnout*: — *Organisational commitment*: — *Retention*: — *Turnover intentions*: A self‐administered questionnaire (developed for this study) *Turnover*: — *Analysis methods*: Chi‐square and independent *t‐*test, generalized estimating equation (GEE) modelling with Bonferroni adjustment for multiple testing
Zhang et al. (2019), Chinese Mainland Longitudinal, non‐randomised control study Quality: GOOD	*Purpose of the study*: To assess the effectiveness of one‐on‐one mentorship programme in reducing the turnover rate of nurses in China *Development of the intervention*: — (not reported) *Aim of the intervention*: To ease new graduate nurses' transition into practice and decrease the turnover rate of new graduate nurses. *Content of the intervention*: Each nurse in the experimental group was assigned a mentor. The mentoring activities were guided by the five functions of mentoring: teach, sponsor, encourage, counsel and befriend. The mentors were provided with a 4‐h orientation programme focused on the development of mentor skills to enable them to be well prepared to support the successful implementation of the mentor programme. NGNs in both groups were given a 3‐week intensive orientation of the hospital *Duration of the intervention*: 12 months	*Setting*: A Tertiary General Hospital, Hangzhou, Zhejiang Province of China *Participants*: New graduate nurses The experimental group (*n* = 239) received the one‐on‐one mentorship programme The control group (*n* = 199) received a basic preceptorship *Follow*‐*up time*: 3 years for each group; turnover rates were calculated annually. The data collection started within the date of employment and ended when the nurse left the job or the study was finished (whichever came first)	*Work engagement*: — *Burnout*: — *Organisational commitment*: — *Retention*: — *Turnover intentions*: — *Turnover*: Turnover rate measured using an electronic file created and stored in the Online Nursing Resource System of the hospital where the resignation of the leaving nurses was marked. The annual turnover rate was the number of nurses who leave the job during one year divided by the number of nurses at the beginning of the year *Analysis methods*: Cox regression analysis

^a^ Follow‐up times have been presented only for the outcomes in interest of this review.

## 3 Results

### 3.1 Studies Included in the Review

The database search yielded 2372 records. A total of 124 duplicates were removed. The reviewers screened 2248 titles, and 25 full texts were retrieved for full screening. Based on the quality appraisal, 10 low‐quality studies were rejected from the review. Other reasons for rejection were participants outside the scope of this review, no intervention or inadequate intervention and high risk of bias. One study was identified through manual search. In total, three studies were included with quality ranging from moderate to good (Figure 1). All included studies were published in international journals in English. One study was conducted in the United States of America, one in Taiwan, China, and one in Chinese Mainland. One was a randomised controlled trial, one a non‐randomised trial and one a cohort study (Table 1).

### 3.2 Description of the Interventions

One intervention was based on a one‐to‐one mentoring strategy (Zhang et al. 2019), one was a digital intervention using supportive text messages (Concilio et al. 2021), and one intervention used mainly educational strategies (Hu et al. 2015) (see Table 1).

The participants of the interventions were entirely NGNs in two studies (Concilio et al. 2021; Zhang et al. 2019), and NGNs as mentees and their mentors in one study (Hu et al. 2015).

The development of the intervention was reported in two studies. A model or a theoretical framework had been used as a basis for development in two interventions (Concilio et al. 2021; Hu et al. 2015). To validate the intervention, an expert panel had been used (Concilio et al. 2021).

The aim of the intervention was expressed in different ways in different studies. As for the main interests of this review, the interventions had aimed to impact NGNs' intention to leave (Concilio et al. 2021) or to decrease NGNs' turnover rate (Zhang et al. 2019). Interventions also aimed, for example, to support NGNs' transition process (Zhang et al. 2019), to impact their work stress (Concilio et al. 2021) or to guide preceptors to discuss problems and issues with the NGNs during the orientation (Hu et al. 2015). More specific aim descriptions are presented in Table 1.

The duration of the shortest intervention had been 6 weeks (Concilio et al. 2021). One intervention had lasted 3 months (Hu et al. 2015), and one 12 months (Zhang et al. 2019).

### 3.3 Outcomes of the Interventions

For evaluating the outcomes, instruments developed for the purpose of this study had been used for measuring turnover intentions in two studies (Concilio et al. 2021; Hu et al. 2015). In one study, the turnover rate was calculated from information obtained from an electronic record created and stored in the Online Nursing Resource System of the hospital (Zhang et al. 2019). Work engagement, burnout, or organisational commitment were not evaluated in any of the studies (Tables 1 and 2).

**TABLE 2:** Effectiveness (effect size, Cohen's *d*) of the interventions.

Original study	Work engagement	Burnout	Organisational commitment	Turnover intentions	Turnover
Concilio et al. (2021)	—	—	—	(no effect)	—
Hu et al. (2015)	—	—	—	−0.58 (medium)	—
Zhang et al. (2019)					−0.36 −0.25 −0.18 (small)

In two studies, the follow‐up measurements were conducted during the implementation of the intervention without follow‐up (Concilio et al. 2021; Hu et al. 2015). In one study (Zhang et al. 2019), the follow‐up time of a 12‐month programme had been three years for each group.

Turnover intentions were evaluated in two studies. A randomised controlled trial by Concilio et al. (2021) included a 6‐week digital intervention consisting of supportive text messages for the experimental group and text messages containing medical facts for the control group. Three variables measuring intention to leave after the intervention (percentage increments, 0%–100%) were analysed: (1) intention to leave current position (ITL1), (2) intention to leave organisation (ITL2) and (3) intention to leave nursing profession (ITL3). The measurement values represented an intention to leave, with lower values indicating a lower intention to leave. The desired outcome was a reduction in these values, reflecting a decrease in turnover intention due to the intervention. After the intervention the mean percentage of intention to leave one's current position was 48.18 (standard deviation (SD) = 31.57) in the experimental group. In the control group, the mean was 45.56 (SD = 53.18). Thus, mean difference (MD) between the groups was 2.62, translating to Cohen's *d* of 0.06, and the *p*‐value for difference between groups was 0.84. After the intervention, the mean percentage of intention to leave one's current organisation was 26.36 (SD = 30.09) in the experimental group. In the control group, the mean was 45.56 (SD = 53.18), MD = −19.20, Cohen's *d* = −0.44, and *p* = 0.16. The mean percentage of intention to leave nursing profession after the intervention was 11.82 (SD = 21.83) in the experimental group and 5.38 (SD = 5.38) in the control group. The MD was 6.44, Cohen's *d* = 0.41, and *p* = 0.20.

The second intervention, conducted by Hu et al. (2015), included a 10‐Minute Preceptor Model (10MP) for the experimental group and a Traditional Preceptor Model (TPM) for the control group. Turnover intentions were enquired using a single‐item measure with scale from 0 to 10, where lower scores indicated a lower intention to leave. The results indicated that in the 10MP group, the mean of turnover intentions was 3.87 (SD = 2.55), while the TPM group had a mean of 5.06 (SD = 1.38). The MD between the groups was −1.19, with a 95% confidence interval (CI) ranging from −1.97 to −0.41. This difference was statistically significant (*p* = 0.003). Furthermore, Cohen's *d* = −0.58 indicated a moderate effect size, suggesting that the 10MP model had a meaningful practical impact in reducing turnover intention among new graduate nurses.

The third study assessed turnover percentage as an outcome. Zhang et al. (2019) conducted a longitudinal, non‐randomised trial that included a one‐to‐one mentorship programme for the experimental group and a basic preceptorship programme for the control group. In the first year after the intervention the mean of turnover rate was 3.77% (SD = 19.08%) in the experimental group. The corresponding figures in the control group were 14.07% (34.33%). The MD was −9.80%, 95% CI from −15.15% to −4.45%, *p* = 0.0004, and Cohen's *d* was −0.36. In the second year after the intervention, the difference between groups remained statistically significant (MD = −5.88%, 95% CI from −10.86% to −0.90%, *p* = 0.021, Cohen's *d* = −0.25), but by the third year, the difference between the groups diluted (MD = −5.44%, 95% CI from −11.93% to 1.05%, *p* = 0.102, Cohen's *d* = −0.18). The findings indicate that the intervention exhibited a modest initial effect, which was not maintained over time, yet it constituted a favourable early response. Notably, this study encompassed a more extended follow‐up period compared to previous studies, thereby enhancing the strength and interpretability of the results.

A summary of the outcomes and effects of the interventions is presented in Table 2.

## 4 Discussion

This systematic review aimed to evaluate the effectiveness of interventions to promote organisational commitment, work engagement and retention and to reduce burnout or turnover of newly graduated nurses. With the criteria set for this review, we found only three interventions, out of which two interventions indicated beneficial effects in reducing NGNs' turnover intentions or actualized turnover. Low turnover intentions can also be viewed as a proxy for high organisational commitment. However, turnover intentions are not the same as actualized turnover, and we reported these results separately.

Two good‐quality studies (Hu et al. 2015; Zhang et al. 2019) showed small and medium effects on lower turnover intentions or actualized turnover. One of them was based on a one‐to‐one mentoring strategy (Zhang et al. 2019), and another was a 10‐Minute Preceptor Model (Hu et al. 2015). Both interventions included education for nurses who were mentors or preceptors and who in that role were supposed to especially support NGNs.

Among other contents, the intervention by Hu et al. (2015) included preceptors' learning on how to structurally communicate, interact and discuss problems and issues with the NGNs. Since these kinds of tasks are not always easy to conduct, training can support the preceptors in their demanding role. Moreover, the preceptors were guided to ask NGNs specific questions twice per day which would take 10 min at a time. Investing in these 20 min of preceptors' time per day may be profitable since it offers an opportunity to grasp the possible problems or issues of NGNs at an early phase. The support by the preceptor or other possible supporters could then be provided in time so that bigger problems could be inhibited, and the NGNs could stay confident that they manage their work. Asking the questions would also produce valuable information on the main problems and issues that NGNs experience in the unit which could then be used in later efforts to attract and retain the nursing workforce. Brook et al. (2019) also identified a period of preceptorship for newly graduated nurses as essential. Organisations' commitment to provide career development to NGNs may potentially lead to positive effects on retention and turnover. Moreover, increases in competence may also result in increased confidence and greater job satisfaction, which is linked with lower turnover (Brook et al. 2019).

A theoretical framework, a model, or a corresponding theoretical framework had been used as a basis for development in two interventions and previous study findings in one intervention. Of the two studies showing association with lower turnover or turnover intentions, Zhang et al. (2019) did not report how the intervention was developed, but Hu et al. (2015) reported that the intervention was developed by the research team based on the five‐step micro‐skill model of clinical teaching. The intervention by Hu et al. (2015) included more than one element and, based on their research findings, it is not possible to precisely determine which of the elements caused the positive effects. However, it can be assumed that using the pedagogical model as a basis could have had positive effects.

Only one study was conducted in a randomised controlled trial design (RCT), although RCT studies are usually considered the ones that best demonstrate the effectiveness of an intervention. A challenge for researchers of the topic in future is to plan robust study designs like RCTs to demonstrate the effectiveness of interventions. In most studies included in this review, the instruments used had been validated. A weakness in studies identified from databases but excluded from this review was the lack of proper statistical testing. While we identified studies with work engagement as an outcome, we had to exclude those due to poor quality and lack of statistical testing. We also found no studies with burnout as an outcome, although almost every fifth NGN has reported extremely high levels of burnout at some point during their first years after graduation and that changes in burnout levels were accompanied by intention to leave the profession (Rudman and Gustavsson 2011).

Even though numerous studies have been carried out on the reasons for turnover of NGNs, only a few interventions have been offered to combat turnover. Interventions have been developed and studied within the framework of residency programmes (Brook et al. 2019). We chose to focus on interventions conducted in situations where the NGNs had been working full‐time in direct patient care with a full salary and excluded residency programmes.

There was no information about total costs of the interventions, or the expected savings generated by an effective intervention in any of the original studies. Counting and reporting these would be important to assist nursing leaders and managers in making decisions on the selection and implementation of interventions to enhance and maintain organisational commitment, work engagement and retention, and to reduce burnout and turnover of NGNs. Problems related to them are global and have implications for the ability of societies to function. Cost‐effective interventions are needed to combat the problem.

### 4.1 Strengths and Limitations

This review used an extensive and systematic search process in online databases, based on a PICO structured question, and with the consultation assistance of academic librarians. The search was reinforced with manual searches. Search terms chosen produced a wide range of hits, and to avoid bias, studies reporting statistically non‐significant results were also included. Two researchers were working independently at first when selecting original studies and when appraising the quality of the studies, which reduces the possibility of subjective selection and appraisal bias.

We decided not to include grey literature, which can be considered as a limitation. Articles published only in English were included, which may cause some bias. We were unable to conduct meta‐analysis due to few and diverse studies.

Two of the studies were conducted in Asian country (China, Taiwan, and Chinese Mainland) and one in Western countries (the USA). The review did not provide information on the impacts of the healthcare system or nursing culture in different contexts. With only three studies, we cannot make any generalizable comparison, but we note that the two Eastern studies used individual‐based methods, while the Western study used a group‐based intervention.

The three studies that were included in this review have rather small sample sizes, with a reported range of 25–400 participants. This limitation is likely to compromise the ability to identify statistically significant differences. This is due to an increase in the standard error, resulting in broader confidence intervals.

## 5 Conclusion

Even though database search yielded many studies, the number of the original studies selected for the review was small. In general, little evidence was found, and information about effective interventions was available only for NGNs' turnover and turnover intentions. Contrary to our expectations, no good quality studies were found investigating interventions to improve the psychological outcomes of work engagement and burnout. The current evidence is thus insufficient to make recommendations for nursing management on interventions to promote NGN's staying at work. To be able to support newly graduated nurses with a high level of impact, more high‐quality research is needed including counting of the costs of the intervention and the savings gained.

## Funding

The authors have nothing to report.

## Disclosure

The authors delare no conflicts of interest.

## Conflicts of Interest

The authors declare no conflicts of interest.

## Supporting information


**Table S1:** Description of the searches in databases.

## Data Availability

The data that supports the findings of this study are available in the Supporting Information of this article.
