# Supplementary material for: Interventions Promoting Work Engagement and Reducing Turnover of Newly Graduated Nurses: A Systematic Review
Source: Nurs Open. 2026 Feb 15;13(2):e70434. doi: 10.1002/nop2.70434 (PMC12907248; doi:10.1002/nop2.70434)
Supplement: Supplementary file 1 — Table S1: Description of the searches in databases. [file NOP2-13-e70434-s001.docx]

**SUPPLEMENTARY TABLE 1** Description of the searches in databases

| **Database**  **Date** | **Search terms** | **Number of records** | **Number of selected studies** |
| --- | --- | --- | --- |
| **Scopus**  4th Apr 2022  Updated 5th Feb 2023 | ( TITLE-ABS-KEY ( "new* graduate* nurse*" ) ) AND ( ( TITLE-ABS-KEY ( ( organizatio* W/2 commit* ) OR ( organisatio* W/2 commit* ) ) ) OR ( TITLE-ABS-KEY ( ( work* OR job ) W/2 engag* ) ) OR ( TITLE-ABS-KEY ( ( work* OR job ) W/2 retention ) ) OR ( TITLE-ABS-KEY ( burnout ) ) OR ( TITLE-ABS-KEY ( ( personnel OR staff OR employee ) W/2 turnover ) ) ) AND ( EXCLUDE ( PUBYEAR , 2011 ) OR EXCLUDE ( PUBYEAR , 2010 ) OR EXCLUDE ( PUBYEAR , 2009 ) OR EXCLUDE ( PUBYEAR , 2008 ) OR EXCLUDE ( PUBYEAR , 2007 ) OR EXCLUDE ( PUBYEAR , 2006 ) OR EXCLUDE ( PUBYEAR , 2005 ) OR EXCLUDE ( PUBYEAR , 2004 ) OR EXCLUDE ( PUBYEAR , 2003 ) OR EXCLUDE ( PUBYEAR , 2002 ) OR EXCLUDE ( PUBYEAR , 1992 ) OR EXCLUDE ( PUBYEAR , 1989 ) ) | 202 | 2 |
| **Cinahl**  4th Apr 2022  Updated 5th Feb 2023 | S23 S3 AND S17 Limiters - Published Date: 20120101-20230205;  Peer Reviewed  Expanders - Apply equivalent subjects  Search modes - Boolean/Phrase  S22 S3 AND S17 Limiters - Published Date: 20030101-20230205;  Peer Reviewed  Expanders - Apply equivalent subjects  Search modes - Boolean/Phrase  S21 S3 AND S17 Limiters - Published Date: 20010101-20230205;  Peer Reviewed  Expanders - Apply equivalent subjects  Search modes - Boolean/Phrase  S20 S3 AND S17 Limiters - Published Date: 20010101-20230205  Expanders - Apply equivalent subjects  Search modes - Boolean/Phrase  S19 S3 AND S17 Limiters - Published Date: 20120101-20230205  Expanders - Apply equivalent subjects  Search modes - Boolean/Phrase  S18 S3 AND S17 Expanders - Apply equivalent subjects  Search modes - Boolean/Phrase  S17 S8 OR S10 OR S13 OR S16 Expanders - Apply equivalent subjects  Search modes - Boolean/Phrase  S16 S14 OR S15 Expanders - Apply equivalent subjects  Search modes - Boolean/Phrase  S15 (personnel OR staff OR employee) N2 turnover  Expanders - Apply equivalent subjects  Search modes - Boolean/Phrase  S14 (MH "Personnel Turnover")  Expanders - Apply equivalent subjects  Search modes - Boolean/Phrase  S13 S11 OR S12 Expanders - Apply equivalent subjects  Search modes - Boolean/Phrase  S12 burnout Expanders - Apply equivalent subjects  Search modes - Boolean/Phrase  S11 (MH "Burnout, Professional+")  Expanders - Apply equivalent subjects    Search modes - Boolean/Phrase  S10 (work* OR job) N2 retention Expanders - Apply equivalent subjects  Search modes - Boolean/Phrase  S9 S3 AND S8 Expanders - Apply equivalent subjects  Search modes - Boolean/Phrase  S8 S4 OR S7 Expanders - Apply equivalent subjects  Search modes - Boolean/Phrase  S7 S5 OR S6 Expanders - Apply equivalent subjects  Search modes - Boolean/Phrase  S6 (work* OR job) N2 engag*  Expanders - Apply equivalent subjects  Search modes - Boolean/Phrase  S5 (MH "Work Engagement")  Expanders - Apply equivalent subjects  Search modes - Boolean/Phrase  S4 organizatio* N2 commit* OR organisatio* N2 commit*  Expanders - Apply equivalent subjects  Search modes - Boolean/Phrase  S3 S1 OR S2 Expanders - Apply equivalent subjects  Search modes - Boolean/Phrase  S2 new* graduate* nurse*  Expanders - Apply equivalent subjects  Search modes - Boolean/Phrase  S1 (MM "New Graduate Nurses")  Expanders - Apply equivalent subjects  Search modes - Boolean/Phrase | 531 | 4 |
| **Cochrane Library**  4th Apr 2022  Updated 5th Feb 2023 | #1 MeSH descriptor: [Nurses] explode all trees  #2 3nurse*):ti,ab,kw (Word variations have been searched)  #3 #1 OR #2  #4 (organizatio* near/2 commit* OR organisatio* near/2 commit*):ti,ab,kw (Word variations have been searched)  #5 MeSH descriptor: [Work Engagement] explode all trees  #6 ((work* OR job) near/2 engag*):ti,ab,kw (Word variations have been searched)  #7 #5 or #6  #8 #4 OR #7  #9 ((work* OR job) near/2 retention):ti,ab,kw (Word variations have been searched)  #10 MeSH descriptor: [Burnout, Professional] explode all trees  #11 (burnout):ti,ab,kw (Word variations have been searched)  #12 #10 OR #11  #13 MeSH descriptor: [undefined] explode all trees  #14 ((personnel OR staff OR employee) near/2 turnover):ti,ab,kw (Word variations have been searched)  #15 #13 OR #14  #16 #3 AND (#8 OR #9 OR #12 OR #15) | 326 | 1 |
| **Medline**  4th Apr 2022  Updated 5th Feb 2023 | exp Nurse Midwives/ or exp Nurse Clinicians/ nurses.sh. 1 or 2 ((organizatio* adj2 commit*) or (organisatio* adj2 commit*)).mp. [mp=title, abstract, original title, name of substance word, subject heading word, floating sub-heading word, keyword heading word, organism supplementary concept word, protocol supplementary concept word, rare disease supplementary concept word, unique identifier, synonyms] work engagement.sh. ((work* or job) adj2 engag*).mp. [mp=title, abstract, original title, name of substance word, subject heading word, floating sub-heading word, keyword heading word, organism supplementary concept word, protocol supplementary concept word, rare disease supplementary concept word, unique identifier, synonyms] 5 or 6 4 or 7 ((work* or job) adj2 retention).mp. [mp=title, abstract, original title, name of substance word, subject heading word, floating sub-heading word, keyword heading word, organism supplementary concept word, protocol supplementary concept word, rare disease supplementary concept word, unique identifier, synonyms] burnout professional.sh. exp Burnout, Professional/ 10 or 11 ((personnel or staff or employee) adj2 turnover).mp. [mp=title, abstract, original title, name of substance word, subject heading word, floating sub-heading word, keyword heading word, organism supplementary concept word, protocol supplementary concept word, rare disease supplementary concept word, unique identifier, synonyms] 3 and 8 3 and 9 3 and 12 3 and 13 14 or 15 or 16 or 17  18 and 2012:2023.(sa year). | 1153 | 1 |
| **JBI**  4th Apr 2022  Updated 5th Feb 2023 | nurses.sh. nurses.mp. [mp=text, heading word, subject area node word, title] 1 or 2 ((organizatio* adj2 commit*) or (organisatio* adj2 commit*)).mp. [mp=text, heading word, subject area node word, title] ((work* or job) adj2 engag*).mp. [mp=text, heading word, subject area node word, title] 4 or 5 ((work* or job) adj2 retention).mp. [mp=text, heading word, subject area node word, title] burnout.sh. burnout.mp. [mp=text, heading word, subject area node word, title] 8 or 9 7 or 10 turnover.sh. ((personnel or staff or employee) adj2 turnover).mp. [mp=text, heading word, subject area node word, title] 12 or 13 3 and 6 3 and 11 3 and 14 15 or 16 or 17 18 and 2012:2023.(sa_year). | 30 | 1 |
| **Medic**  4th Apr 2022  Updated 5th Feb 2023 | "Nurses" (asiasanat)  "Work Engagement" (asiasanat)  "Burnout, Professional" (asiasanat)  "Nurses" (asiasanat) AND "Work Engagement" OR "Burnout, Professional" 2012 - 2023 väit.,alkup.tutk.,kats.art.  sairaanhoitaja* OR kätilö* OR hoitaja* AND "työhön sitoutumi* OR "työn imu*" OR "työpaikassa pysym*" OR "pitovoim*" OR työuupum* OR "alalta poistum*" 2012 - 2023 väit.,alkup.tutk.,kats.art. | 30 | 0 |
